# Case management to increase quality of life after cancer treatment: a randomized controlled trial

**DOI:** 10.1186/s12885-017-3213-9

**Published:** 2017-03-28

**Authors:** Nathalie Scherz, Irène Bachmann-Mettler, Corinne Chmiel, Oliver Senn, Nathalie Boss, Katarina Bardheci, Thomas Rosemann

**Affiliations:** 1 Institute of Primary Care, University of Zurich, University Hospital Zurich, Zurich, Switzerland; 2 Arud, Centres for Addiction Medicine, Zurich, Switzerland

**Keywords:** Cancer, case management, survivors, quality of life, Self-Efficacy, self care, health behaviour

## Abstract

**Background:**

Case management has been shown to be beneficial in phases of cancer screening and treatment. After treatment is completed, patients experience a loss of support due to reduced contact with medical professionals. Case management has the potential to offer continuity of care and ease re-entry to normal life. We therefore aim to investigate the effect of case management on quality of life in early cancer survivors.

**Methods:**

Between 06/2010 and 07/2012, we randomized 95 patients who had just completed cancer treatment in 11 cancer centres in the canton of Zurich, Switzerland. Patients in the case management group met with a case manager at least three times over 12 months. Patient-reported outcomes were assessed after 3, 6 and 12 months using the Functional Assessment of Cancer Therapy (FACT-G) scale, the Patient Assessment of Chronic Illness Care (PACIC) and the Self-Efficacy scale.

**Results:**

The change in FACT-G over 12 months was significantly greater in the case management group than in the control group (16.2 (SE 2.0) vs. 9.2 (SE 1.5) points, *P* = 0.006). The PACIC score increased by 0.20 (SE 0.14) in the case management group and decreased by 0.29 (SE 0.12) points in the control group (*P* = 0.009). Self-Efficacy increased by 3.1 points (SE 0.9) in the case management group and by 0.7 (SE 0.8) points in the control group (*P* = 0.049).

**Conclusions:**

Case management has the potential to improve quality of life, to ease re-entry to normal life and to address needs for continuity of care in early cancer survivors.

**Trial registration:**

The study has been submitted to the ISRCTN register under the name “Case Management in Oncology Rehabilitation” on the 12th of October 2010 and retrospectively registered under the number ISRCTN41474586 on the 24th of November 2010.

**Electronic supplementary material:**

The online version of this article (doi:10.1186/s12885-017-3213-9) contains supplementary material, which is available to authorized users.

## Background

Cancer and its associated multimodal therapies have long-term negative effects on quality of life. Many survivors experience declines in physical, psychological, and social functioning and perceived role function, which significantly impacts their careers [1–4]. This risk of decline strongly varies between patients and is greater in socially isolated patients [5, 6]. Consequently, patients need tailored support when they return to everyday life after undergoing cancer treatment. With the trend of decentred outpatient care, patients must meet with numerous different healthcare providers. Therefore, despite the fact that social counselling, psycho-oncological therapy and opportunities to increase physical fitness are widely offered, the needs of patients frequently cannot be met [7, 8]. The reasons are multifaceted: after undergoing treatment, many patients struggle to identify their needs for re-entry into everyday life, and they lack energy to organize their rehabilitation measures, which are also uncoordinated [9].

These challenges have many similarities with those encountered by patients with chronic medical conditions. It therefore seems likely that the features of the chronic care model could address many of the mentioned burdens [10]. Our hypothesis was that case management (CM) could be one way to address behavioural and psychosocial issues better than usual care. A case manager can assess patients’ needs; identify barriers; inform patients about existing rehabilitation programmes; ensure coordination between the patient, physicians and other care providers; and thus offer care for cancer survivorship in accordance with the chronic care model [11]. Moreover, a case manager can aim at empowering patients in organizing targeted measures, thus promoting self-management skills, self-efficacy and lifestyle modifications [12, 13]. These interventions may enable cancer survivors to cope with the long-term consequences of cancer and thus increase health-related quality of life and may support employability.

CM or similar models of advanced nursing (such as patient navigation, pivot nursing or contact nursing) have been assessed for the phases of cancer screening and cancer treatment, showing mixed results [14, 15]. Recent studies investigating patients during cancer treatment showed a decrease in emergency room visits and lower cancer-related medical costs [16, 17]. However, no studies are available on the effects of CM on quality of life after completion of cancer treatment. To assess this, we aimed at comparing the effect of CM versus usual care on the quality of life in early cancer survivors.

## Methods

The rationale, design and methods of the study have been reported previously [18]. The study protocol was approved by the ethics committee of the canton of Zurich on the 10th of May 2010 (Ref. KEK-ZH-NR: 2009–0145/1).

### Patient population

The inclusion criteria were 18 years old or older, completion of a curatively intended cancer treatment (chemotherapy, radiotherapy and/or surgery), expected survival of at least 1 year, increased distress scale (≥3) according to the commonly used Distress Thermometer [19], and need of and intention to undertake rehabilitation according to patient’s perspective. Due to difficulties in patient recruitment, two inclusion criteria had to be altered from the protocol: patients with all types of cancer were eligible instead of patients with breast cancer only, and the initially required Distress Thermometer between 3 and 7 was extended to an upper limit of 9. Patients with a Distress Thermometer >7 (high distress) were immediately contacted by the study nurse to assess if further referral to psychiatric support was needed. Following this brief assessment, only patients reporting a temporary and not persistent severe distress were included in the study. The exclusion criteria were treatment completed more than 1 month ago, metastasis and/or advanced stage disease, cancer relapse during study, palliative treatment, insufficient knowledge of German to participate in counselling and evaluation, or severe psychiatric disease. Patients with a relapse (3 in the CM group and 2 in the usual care (UC) group) were excluded from the intention-to-treat analysis, but CM was continued for ethical reasons. Nurses and physicians in 11 cancer centres in the region of Zurich informed eligible patients about the study and with their permission transmitted contact data to the study nurse, who asked the patients for written informed consent.

### Randomization

The randomization of the list was performed by a scientist at the Institute of Primary Care not involved in the study. Patients were randomized with computer generated numbers, in block sizes of 2 and 4, stratified according to the type of cancer. The study nurse attributed the next following number to each patient and opened the sealed numbered envelope containing the randomization allocation.

### Intervention

Details on the intervention concept have been reported in the study protocol [18]. The five case managers were nurses specializing in oncology, skilled in discussing patients’ problems in an empathic way and able to offer resource-oriented, self-empowering motivational counselling. During the first 3 months, they met with the patients at least three times to establish a relationship, assess needs, and generate an action plan. By means of a standardized structured electronic tool, the following items were addressed: past medical history (in the first interview), current mental state, stresses and challenges, influencing factors, resources, goals and measures. The case managers provided information on available services and therapies and helped organize appointments. In the following months, they performed telephone follow-ups according to individual patients’ needs. The case managers were available on demand during office hours. The entire intervention lasted up to 12 months with a final concluding interview.

### Outcome measures

The study nurse sent out questionnaires to the patients with a stamped response envelope at baseline, three, six and 12 months. Non-responding patients were contacted once by phone. The primary outcome was health-related quality of life at 12 months. It was measured with the Functional Assessment of Cancer Therapy-General (FACT-G) questionnaire [20]. The FACT score rates the health-related quality of life in patients treated for cancer. The score sums up to a total ranging from zero to 108 points, where a higher score indicates better quality of life. The secondary outcomes were Self-Efficacy measured with the Jerusalem & Schwarzer questionnaire [21]. The result can range between 10 and 40 with a higher score indicating more self-efficacy (meaning having a stronger belief in one’s competence to cope with a broad range of stressful or challenging demands). Accordance of received care with the chronic care model was evaluated with the Patient Assessment of Chronic Illness Care (PACIC) [22]. The PACIC Score can range between one and five, with a higher score indicating a better accordance of care with the chronic care model. The PACIC was slightly adapted to be used in the context of rehabilitation for cancer survivorship instead of care for chronic illness. Patients were asked about their employability and family status. Health utilization as well as other support measures or services utilized in the last 3 months were assessed. Finally, patients were asked if they made conscious changes in their lives with respect to their physical activity, diet, work, and relaxation practice.

### Safety issues

As stated in the informed consent patient form, serious risks or undesired effects of the intervention or the assessment by questionnaires have not been described in the literature. There are no specific risks related to the study. The study is being conducted in accordance with medical professional codex and the Helsinki Declaration as of 1996 as well as Data Security Laws. Study participation of patients is voluntary and can be cancelled at any time without provision of reasons and without negative consequences for their future medical care.

### Statistical analysis

All analyses were performed according to the intention-to-treat principle and therefore used the latest available measurement for missing values (last observation carried forward). The sum of the FACT-G items was calculated allowing three missing items per domains and five of the overall items (out of 27). The average value for the PACIC and Self-Efficacy items was calculated. Three missing items (out of 20) were allowed for the construction of the PACIC and three (out of 10) for the construction of the Self-Efficacy questionnaire. Residual missing values were replaced by the mean of the remaining values. For the other outcomes, the missing values are mentioned in the result tables. Continuous outcomes were compared with the Wilcoxon Rank-Sum and with the unpaired t-test. Categorical variables were compared with the Fisher’s exact and chi-square test. Changes in proportions over time were compared with the McNemar test and change of continuous variable with the paired t-test. To account for differences in baseline FACT-G, PACIC and Self-Efficacy, a linear regression analysis of the 12 month values adjusted for baseline as well as for Distress Thermometer was performed. Adjusted means for each group and adjusted differences in change in and between groups were computed. The effect size was computed by dividing the adjusted difference in change by the pooled baseline standard deviation. To assess the trend of the time course and of the intervention on outcomes within groups and between groups, a mixed effect linear regression for repeated measures was performed including an interaction term (time*group) as a covariate. We controlled for a potential cluster effect of treatment centre and coaches on the primary outcome by comparing the regression model with a hierarchical model clustered for cancer treatment centre and coaches using the likelihood-ratio test. All of the reported *P* values were 2-sided. A *P* < 0.05 was considered statistically significant. All of the analyses were performed with StataCorp. 2013. Stata Statistical Software: Release 13. College Station, TX, USA: StataCorp LP.

## Results

**Fig. 1:**
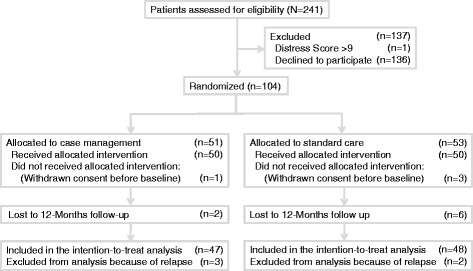
CONSORT Flow-Chart

Of the 104 patients who agreed to participate, 51 were allocated to CM and 53 to UC. One patient in the CM group and three in the UC group withdrew consent before completing the baseline questionnaires and thus could not be included in the analysis. Because of organizational and financial reasons and difficulties with recruiting, we could not further increase our sample after these withdrawals. We excluded three patients in the CM and two in the UC group from the analysis because of cancer recurrence. Ultimately, 47 patients in the CM and 48 in the UC group were included in the analysis. Two patients in the CM and three in the UC group were lost to follow-up at 3 months, one more in the UC group at 6 months and three more at 12 months (Fig. 1). These patients declined to complete further questionnaires or moved without giving their new address and phone number to the study nurse (but did not withdraw consent).

The baseline characteristics of the study population are presented in Table 1. Overall, 92% of the patients were female, and the mean age was 50 years. The FACT-G was significantly different between the groups at baseline, with a mean (SD) of 67.9 (16.0) points in the CM and 74.9 (14.3) in the UC group (*P* = 0.03). The Distress Thermometer was significantly higher in the CM than in the UC group (6.2 (SD 1.46) vs. 5.6 (SD 1.45) *P* = 0.04).

**Table 1:** Baseline Characteristics

	CM *n* = 47		UC *n* = 48		
	No.	(%)	No.	(%)	*P*
Female	41	(87)	46	(96)	.16
Family Status					.77
Single	12	(26)	12	(25)	
Married	21	(45)	26	(54)	
Widowed	1	(2)	1	(2)	
Divorced/Separated	13	(28)	9	(19)	
Foreigner	12	(26)	11	(23)	.81
Cancer localization					1
Breast	35	(75)	35	73	
Colorectal	4	(9)	5	(10)	
Lungs	1	(2)	0		
Hodgkin lymphoma	1	(2)	0		
Uveal Melanoma	0		1	(2)	
Non-Hodgkin lymphoma	2	(4)	3	(6)	
Ovarian	2	(4)	3	(6)	
Prostate	1	(2)	0		
Cervix	0		1	(2)	
Larynx	1	(2)	0		
Cancer therapy					
Chemotherapy	45	(96)	44	(92)	.68
Radiotherapy	39	(83)	36	(75)	.45
Hormonal therapy	24	(51)	28	(58)	.54
Surgery	43	(91)	44	(92)	1
Age, Mean	49.6		50.8		.56
SD	11.0		8.9		
School years, Mean	14.8		13.8		.17
SD	4.1		3.3		
Patient-reported Outcomes					
FACT-G, Mean	67.9		74.9		.03
SD	16.0		14.3		
Self-Efficacy, Mean	26.8		28.2		.19
SD	4.9		5.5		
PACIC, Mean^a^	2.33		2.61		.13
SD	0.80		0.97		
Distress Thermometer, Mean^b^	6.2		5.6		.04
SD	1.46		1.45		

*CM* case management, *UC* usual care, *SD* standard deviation, *FACT-G* functional assessment of cancer therapy, *ICR* interquartile range, *PACIC* patient assessment of chronic illness care

^a^3 missing in CM, 1 missing in UC

^b^1 missing in CM, 1 missing in UC

### Primary outcome

**Fig. 2:**
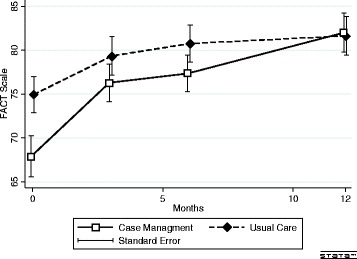
Crude FACT-G scale mean over time. The repeated measure mixed model regression analysis showed a significant trend for time overall (*P* < 0.001) and a significant trend for time* group (*P* = 0.002)

In comparison to baseline, both groups had a significant increase of FACT-G over 12 months (both *P* < 0.001) (Fig. 2). There was no difference in FACT-G between the groups at 12 months. The increase in the CM group was significantly greater than the increase in the UC group (mean (SE) 16.2 (2.0) versus 9.2 (1.5) points (*P* = 0.006), with a mean difference in change between groups of 7.0 (2.5) points (*P* = 0.006). The increase in the FACT-G after 12 months in the CM compared to the UC group remained significantly higher when adjusted for differences in baseline FACT-G and Distress Thermometer (Table 2). The effect size (Cohen d) was moderate, with a value of 0.43. The cluster analysis showed no cluster effect for the cancer treatment centre or for the coach on the FACT-G (*P* < 0.0001).

**Table 2:** Crude and Adjusted 12 Month Outcomes, Changes within and between Groups

	12 Months				Change within group				Difference in Change		
	CM *n* = 47		UC *n* = 48		CM *N* = 47		UC *n* = 48				
	Mean	SE	Mean	SE	Mean	SE	Mean	SE	Mean	SE	*P*
FACT-G crude	82.0	2.2	81.6	2.2	16.2	2.0	9.2	1.5	7.0	2.5	.006
FACT-G adjusted for Baseline	84.3	1.7	79.3	1.6	15.2	1.7	10.2	1.7	5.0	2.4	.04
FACT-G adjusted for Baseline + Distress	84.8	1.7	78.8	1.6	15.7	1.7	9.7	1.6	6.0	2.4	.01
Self-Efficacy crude	29.9	0.7	28.9	0.9	3.1	0.9	0.7	0.8	2.4	1.2	.049
Self-Efficacy adjusted for Baseline	30.2	0.7	28.6	0.7	2.7	0.7	1.1	0.7	1.6	1.0	.13
Self-Efficacy adjusted for Baseline + Distress	30.5	0.7	28.4	0.7	3.0	0.7	0.8	0.7	2.1	1.0	.046
PACIC crude	2.56	0.12	2.32	0.13	0.20	0.14	−0.29	0.12	0.49	0.18	.009
PACIC adjusted for Baseline	2.60	0.12	2.25	0.11	0.13	0.11	−0.22	0.11	0.34	0.16	.034
PACIC adjusted for Baseline + Distress	2.62	0.12	2.24	0.12	0.14	0.12	−0.24	0.12	0.37	0.17	.03

*CM* case management, *UC* usual care, *SE* standard error, *FACT-G* functional assessment of cancer therapy, *PACIC* patient assessment of chronic illness care

### Secondary outcomes

**Fig. 3:**
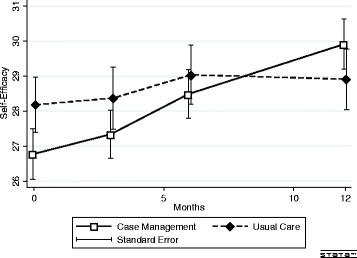
Crude Self-Efficacy mean over time. The repeated measure mixed model regression analysis showed a significant trend for time overall (*P* < 0.001) and for time*group (*P* = 0.002)

Self-Efficacy increased in the CM group (29.9 (SE 0.7) vs. 26.8 (SE 0.7), *P* = 0.001) and showed no significant change over time in the UC group (28.9 (SE 0.9) vs. 28.2 (SE 0.8), *P* = 0.38) (Fig. 3). Thus, the change was significantly higher in the CM than in the UC group (3.1 (SE 0.9) versus 0.7 (SE 0.8) points, *P* = 0.049).

**Fig. 4:**
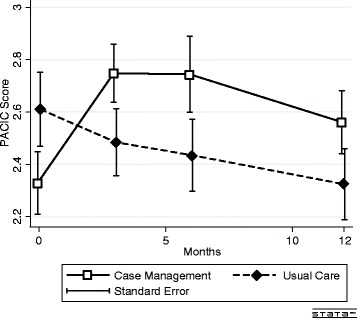
Crude PACIC scale mean over time. The repeated measure mixed model regression analysis in the UC group showed a negative trend for time (*P* = 0.005)

The PACIC score decreased continuously over the 12 months in the UC group from 2.61 (SE 0.14) to 2.32 (SE 0.13) points (*P* = 0.02) (Fig. 4). In the CM group, PACIC initially increased over 3 months (2.32 (SE 0.11) vs. 2.71 (SE 0.10), (*P* = 0.0003) and then tended to decrease again over the remaining time ((2.56 (SE 0.12) vs. 2.75 (SE 0.11), *P* = 0.06). There were no differences between the groups in the self-reported changes in diet, physical activity, relaxation practice (see Additional file 1: Table S1) or in employability (Table 3).

**Table 3:** Working status and sick day leaves over time

	Baseline					3 Months					6 Months					12 Months				
	CM *n* = 47		UC *n* = 48		*P*	CM *n* = 45		UC *n* = 46		*P*	CM *n* = 45		UC *n* = 45		*P*	CM *n* = 45		UC *n* = 42		*P*
	n	(%)	n	(%)		n	(%)	n	(%)		n	(%)	n	(%)		n	(%)	n	(%)	
Working status					.24					.20					.36					.42
Working 100%	14	(30)	18	(38)		10	(22)	14	(30)		9	(20)	11	(24)		6	(13)	5	(12)	
Working part time	19	(40)	20	(42)		17	(36)	22	(48)		21	(47)	24	(53)		20	(44)	27	(64)	
Housewife/−husband	6	(13)	3	(6)		7	(16)	4	(9)		6	(13)	4	(9)		7	(16)	4	(10)	
Unemployed	0		3	(6)		1	(2)	3	(7)		0		2	(4)		3	(7)	2	(5)	
Disability pension	0		0			1	(2)	0			0		0			0		0		
Retired	8	(17)	4	(8)		9	(20)	3	(7)		9	(20)	4	(9)		9	(20)	4	(10)	
Sick day leave number of responses	37		42			36		43			37		42			34		39		
Patients with sick day leaves	32 (68)		34 (70)		.56	16 (34)		21 (44)		.82	19 (40)		14 (30)		.12	13 (22)		9 (23)		1
Sick day leaves, Median IQR	48 15;60		41 20;60		.58	13 0;51		1 0;40		.44	1 0;14		0 0;3		.16	0 0;2		0 0;1		.67

*CM* case management, *UC* usual care, *IQR* interquartile range

There were no differences between the groups in the amount of contact with a physician or the number of medical and unplanned visits (see Additional file 2: Table S2). Similarly, there were no differences in hospital or rehabilitation clinic stay and median length of stay. Significantly more patients in the UC group mentioned having met a physiotherapist at 3 months (20 out of 45 versus 10 out of 46 in the CM group, *P* = 0.04) and a lymphatic drainage therapist at 6 months (15 out of 46 vs. 5 out of 45 in the CM group, *P* = 0.02). Significantly more patients in the CM group mentioned having had support for childcare at 6 and 12 months (7 out of 45 in the CM group versus none in the UC group, *P* = 0.012). There were no differences between the use of other therapies (diet counselling, psychologist, medical training therapy, stress reduction therapy, courses to support quality of life, self-help groups, breast care nurse, stoma care nurse, other nurse), services (national cancer association, patient counselling office, social services, human resources, internet) or support (help for housekeeping, for childcare, for personal hygiene) (see Additional file 3: Table S3). Less than 10% of the patients used opiates, sleeping pills, sedative analgesics or benzodiazepines, with no difference between the groups at any point in time. Between 14 and 27% of all patients used antidepressant drugs, without a significant difference between the groups at any point in time (see Additional file 4: Table S4).

In both groups, the number of patients working full time decreased significantly over 12 months (CM 14 to 6, *P* = 0.039, UC 18 to 5, *P* < 0.001) with a significant increase of patients working part time in the UC group (20 to 27, *P* = 0.006) but not in the CM group (19 to 20, *P* = 0.55) (Table 3). The number of patients with sick day leaves decreased significantly in both groups (CM 32 to 13, *P* < 0.001, UC 34 to 9, *P* < 0.001), with no difference between the groups at any point in time.

## Discussion

Our study on CM among early cancer survivors showed that, compared to UC, CM leads to a greater increase in quality of life (FACT-G), higher Self-Efficacy scores, and health care that is more in accordance with the chronic care model (PACIC).

Although our study was not able to show a significant absolute difference between the groups in the FACT-G after 12 months, we still consider our main outcome as highly relevant for two reasons: 1) the difference in change in quality of life, adjusted for relevantly lower FACT-G scores at baseline and other differences in baseline characteristics, was not only highly significant but also 2) highly relevant: the CM group showed a difference (increase) in the FACT-G score of 7 points. This value is greater than the minimal difference considered significant of three, for which the study was powered [18, 23].

A former study performed among early cancer survivors shared similarity with our intervention and showed an effect on mood and cancer-related concerns but specifically targeted an underserved population [24]. A self-selected trial of phone-based case management could decrease cost, but quality of life was not assessed [17]. Overall, a meta-analysis showed psycho-oncologic interventions to be associated with a small-to-medium positive effect on quality of life [25]. However, to our knowledge, our study is the first to examine the effect of CM on the quality of life of early cancer survivors.

Several reasons can explain the beneficial effect of CM in this setting. First, the case manager provided important information on long-term symptoms and on [26] available services and therapies. This is in accordance with previous data, showing that cancer patients would value additional information on many topics, including chronic symptoms, handling long-term treatment in everyday life, financial issues and preparation for returning to work [27, 28]. A former study showed that a patient’s reported quality of life correlated with the access to helpful information. Second, in accordance with the chronic care model, the case manager offered a continuity of care when appointments for treatment ceased and medical follow up visits were less frequent [11]. Thus, CM provided a type of substitution for decreasing healthcare support. This finding is reflected in our data by an increase of the PACIC in the first 3 months in the CM group, opposed to the overall decrease of PACIC throughout the 12 months of follow up in the UC group (Fig. 4). Third, the case manager offered support to cope with the psychological issues of the re-entry phase to normal life [9]. The assessment helped patients realize that they were facing new needs and challenges. The motivational counselling empowered self-management and gave them tools to face the upcoming challenges [12]. These approaches are reflected in the greater increase of the Self-Efficacy scale in the CM group. Alternatively, patients’ needs were more orientated to information, continuity and support to cope with new challenges. Because we observed almost no changes in the self-reported use of supporting services, medical training therapy, physiotherapy and psychotherapy, it seems unlikely that the effect of the CM would have been mediated by an increased use of these offers. Furthermore, our study showed no effect on self-reported physical activity, diet, relaxation practice or employability. This finding was not surprising because the case manager’s intervention was not focused on convincing patients to adhere to a fitness programme or to return to work. The effects of CM on quality of life and Self-Efficacy are therefore not explained by increases in health care use but rather by the psychoeducational intervention of CM, a finding that is consistent with former studies showing that psychoeducational interventions have positive effects on cancer patients [25].

Our study has several potential limitations. The main limitation is the difference in baseline FACT-G between the groups. We cannot exclude that the greater increase of FACT-G in the CM group reflects the natural history of cancer patients with worse health-related quality of life. Another possible limitation is the self-selection process of the patients. Because 137 of 241 eligible patients declined to participate, we cannot conclude that the CM approach would have positive effects on all patients. It is possible that this effect only occurs in patients with some affinity for CM. Several studies showed a large heterogeneity in the quality of life of cancer survivors, resulting in completely different needs for the re-entry phase [29, 30]. Most mentioned that the reason for declining to participate in our study was a lack of need for additional support. More research is needed to determine how to select patients likely to benefit from such a service. Furthermore, a frequent limitation of CM interventions is that the effect of the case manager’s own personality remains unclear. This bias can be excluded in our study, as we had five different case managers and could not detect any cluster effects. This finding reflects the standardized approach of our intervention and suggests its transferability to other settings. A notable strength of our study is that CM, as practiced in our intervention, constitutes a new practice in Switzerland. Therefore, we were able to compare the effect of CM versus UC in early cancer survivors, a setting in which it had never been tested and which is not possible in other countries where similar nurse-led follow-ups have previously been implemented [15, 31].

## Conclusion

CM, in which a trained nurse assesses needs, offers information, and provides empowering support, eases re-entry to normal life and addresses needs for continuity of care in early cancer survivors. This is a practical approach to coordinate existing rehabilitation programmes in the fragmented oncological healthcare system and to address the heterogenic needs of cancer survivors. More research is needed to identify the patients who can benefit most from such interventions.

## Additional files

Additional file 1: **Table S1.** Patients’ reported lifestyle changes (PDF 792 kb)

Additional file 2: **Table S2.** Hospital stay, rehabilitation stay, medical visits (PDF 117 kb)

Additional file 3: **Table S3.** Use of therapies, counselling, support (PDF 357 kb)

Additional file 4: **Table S4.** Patients taking pain psychoactive drugs. (PDF 428 kb)

## Abbreviations

CM: Case management
FACT-G: Functional assessment of cancer therapy-general
IQR: Interquartile range
PACIC: Patient assessment of chronic illness care
SD: Standard deviation
SE: Standard error
UC: Usual care
