# Supplementary material for: Case management to increase quality of life after cancer treatment: a randomized controlled trial
Source: BMC Cancer. 2017 Mar 28;17:223. doi: 10.1186/s12885-017-3213-9 (PMC5368904; doi:10.1186/s12885-017-3213-9)
Supplement: Supplementary file 1 — Table S1. Patients’ reported lifestyle changes (PDF 792 kb) [file 12885_2017_3213_MOESM1_ESM.pdf]

Table S1. Patients' reported lifestyle changes																					
	Baseline					3 Months					6 Months					12 Months					
	CM n=47		UC n=48		*P*	CM n=45		UC n=46		*P*	CM n=45		UC n=45		*P*	CM n=45		UC n=42		*P*	
	n	(%)	n	(%)		n	(%)	n	(%)		n	(%)	n	(%)		n	(%)	n	(%)		
Physical activity	14	(30)	21	(44)	.20	23	(51)	28	(61)	.40	27	(60)	24	(53)	.67	25	(56)	18	(43)	.28	
Diet	15	(32)	21	(44)	.29	26	(58)	18	(39)	.10	25	(56)	20	(44)	.40	24	(53)	22	(52)	1	
Stress reduction measures	14	(30)	18	(38)	.52	19	(42)	22	(48)	.68	18	(40)	21	(47)	.67	21	(47)	18	(43)	.83	
Work	8	(17)	3	(6)	.12	12	(27)	8	(17)	.32	12	(27)	10	(22)	.81	10	(22)	12	(29)	.62	
Other	3	(6)	3	(6)	1	5	(11)	2	(4)	.27	7	(16)	3	(7)	.32	6	(13)	4	(10)	.74	
CM: Case Management; UC: Usual Care; IQR: interquartile range																					
