# Supplementary material for: Case management to increase quality of life after cancer treatment: a randomized controlled trial
Source: BMC Cancer. 2017 Mar 28;17:223. doi: 10.1186/s12885-017-3213-9 (PMC5368904; doi:10.1186/s12885-017-3213-9)
Supplement: Supplementary file 2 — Table S2. Hospital stay, rehabilitation stay, medical visits (PDF 117 kb) [file 12885_2017_3213_MOESM2_ESM.pdf]

Table S2. Hospital stay, rehabilitation stay, medical visits												
	Baseline			3 Months			6 Months			12 Months		
	CM	UC	*P*	CM	UC	*P*	CM	UC	*P*	CM	UC	*P*
	n(%)	n(%)		n(%)	n(%)		n(%)	n(%)		n(%)	n(%)	
**Inpatient stay**												
Stayed at the hospital	12(26)	14(29)	.81	2(4)	5(11)	.44	7(16)	1(2)	.06	5(11)	3(7)	.72
Stayed in rehabilitation	1(2)	2(4)	1	6 (14)	1(2)	.05	1(2)	2(5)	1	0	0	1
**Medical visits during the last 3 months, Median (IQR)**												
Went to GP	24(51)	22(46)	.68	26(60)	26(58)	1	24(53)	27(60)	.67	24(53)	23(55)	.53
Number of visits	1	0	0.33	1	1	.96	1	1	.52	0.5	1	.60
(IQR)	(0;2)	(0;1)		(0;2)	(0;2)		(0;1)	(0;2)		(0;2)	(0;2)	
Went to oncologist	39(83)	40(83)	1	34(76)	31(67)	.49	30(67)	22(49)	.14	20(45)	19(45)	1
Number of visits	3	4.5	.19	1	1	.4	1	0	.03	0	0	.46
(IQR)	(1;5)	(1;7)		(0.5;2.5)	(0;2)		(0;2)	(0;1)		(0;1)	(0;1)	
Went to Gastroenterologist	3(6)	1(2)	.36	1(2)	4(9)	.36	3(7)	4(9)	1	2(4)	4(10)	.42
Number of visits	0	0	1	0	0	.18	0	0	.70	0	0	.35
(IQR)	(0;0)	(0;0)		(0;0)	(0;0)		(0;0)	(0;0)		(0;0)	(0;0)	
Went to Gynecologist	19(40)	27(56)	.15	25(56)	26(57)	1	21(47)	27(60	.30	20(44)	26(62)	.13
Number of visits	0	1	.25	1	1	.78	0	1	.18	0	1	.12
(IQR)	(0;1)	(0;1)		(0;1)	(0;1)		(0;1)	(0;1)		(0;1)	(0;1)	
Went to Psychiatrist	15(32)	15(31)	1	19(42)	12(26)	.13	18(40)	10(22)	.11	12(27)	8(19)	.45
Number of visits	0	0	.94	0	0	.69	0	0	.12	0	0	.69
(IQR)	(0;3)	(0:2)		(0;0)	(0;0)		(0;2)	(0;0)		(0;0)	(0;0)	
Patients with unplanned visits	22(47)	19(40)	.54	22(47)	17(35)	.30	13(28)	17(35)	.51	18(38)	19(40)	1
Unplanned visits	0	0	.54	0	0	.25	0	0	.44	0	0	.90
(IQR)	(0;1)	(0;1)		(0;2)	(0;1)		(0;0)	(0;0)		(0;1)	(0;1)	
