# Supplementary material for: Case management to increase quality of life after cancer treatment: a randomized controlled trial
Source: BMC Cancer. 2017 Mar 28;17:223. doi: 10.1186/s12885-017-3213-9 (PMC5368904; doi:10.1186/s12885-017-3213-9)
Supplement: Supplementary file 3 — Table S3. Use of therapies, counselling, support (PDF 357 kb) [file 12885_2017_3213_MOESM3_ESM.pdf]

Table S3. Use of therapies, counselling, support

	Baseline			3 Months			6 Months			12 Months		
	CM n=47	UC n=48	*P*	CM n=45	UC n=46	*P*	CM n=45	UC n=45	*P*	CM n=45	UC n=42	*P*
	n(%)	n(%)		n(%)	n(%)		n(%)	n(%)		n(%)	n(%)	
Therapies, counselling, support												
Physiotherapy	12(26)	19(40)	.19	10(22)	20(44)	.04	14(31)	17(38)	.66	17(38)	13(30)	.51
Diet counselling	4 (9)	6(13)	.74	2(4)	2(4)	1	2(4)	1(2)	1	2(4)	1(2)	1
Lymphatic drainage	7 (15)	14(30)	.14	7(16)	13(28)	.21	5(11)	15(33)	.02	7(16)	11(26)	.29
Psychologist	18(38)	18(38)	1	18(40)	14(30)	.39	18(40)	12(27)	.26	12(27)	7(16)	.30
Sport therapy	4(9)	4(8)	1	7(16)	8(17)	1	8(18)	8(18)	1	5(11)	7(15)	.55
Stress reduction therapies	4(9)	8(17)	.36	8(18)	9(20)	1	8(18)	9(20)	1	8(18)	5(12)	.55
Patient education	3(6)	6(13)	.49	1(2)	3(7)	.62	1(2)	3(7)	.62	4(9)	4(9)	1
Self-help groups	2(4)	2(4)	1	3(7)	1(2)	.62	2(4)	4(9)	.68	1(2)	1(2)	1
National cancer association	7(15)	9(19)	.78	14(31)	8(17)	.15	3(7)	10(22)	.07	6(13)	6(14)	1
Patient counselling office	1(2)	1(2)	1	0	1(2)	1	0	1(2)	1	0	1(2)	.48
Social services	0(0)	3(6)	.24	3(7)	1(2)	.36	2(4)	1(2)	1	0	1(2)	.2
Nursing services	8(17)	9(19)	1	2 (4)	1(2)	.62	0	0	1	0	1(2)	.48
Breast care nurse	4(9)	4(8)	1	2(4)	1(2)	.62	0	1(2)	1	0	0	1
Stoma care nurse	1(2)	1(2)	1	1(2)	1(2)	1	1(2)	1(2)	1	0	1(2)	.48
Human resources	2(4)	3(6)	1	3(7)	4(9)	1	1(2)	3(7)	.62	1(2)	1(2)	1
Internet	16(34)	18(38)	.83	11(24)	16(35)	.36	8(18)	8(18)	1	5(11)	6(14)	.75
Other therapy, counselling, support	8(17)	10(21)	.80	19(42)	5(11)	.001	8(18)	5(11)	.55	9(20)	5(12)	.39
No therapy, counselling or support	6(13)	3(6)	.32	6(13)	5(11)	.76	6(13)	5(11)	1	12(27)	6(14)	.19
Help for everyday life												
Help for housekeeping	16(34)	24(50)	.15	11(24)	11(24)	1	10(22)	10(22)	1	8(18)	7(17)	1
Help for childcare	11(23)	4(8)	.05	6(13)	3(7)	.32	7(16)	0	.012	7(16)	0	.012
Help for personal hygiene	2(4)	1(2)	.61	0	0		0	0		0	0	
Other help	3(6)	2(4)	.68	0	0		1(2)	1(2)	1	1(2)	0	1
No help	28(60)	24(50)	.41	32(71)	32(70)	1	30(67)	35(78)	.35	35(78)	34(81)	.80
