# Supplementary material for: Case management to increase quality of life after cancer treatment: a randomized controlled trial
Source: BMC Cancer. 2017 Mar 28;17:223. doi: 10.1186/s12885-017-3213-9 (PMC5368904; doi:10.1186/s12885-017-3213-9)
Supplement: Supplementary file 4 — Table S4. Patients taking pain psychoactive drugs. (PDF 428 kb) [file 12885_2017_3213_MOESM4_ESM.pdf]

Table S4. Patients taking pain and psychoactive drugs

	Baseline					3 Months					6 Months					12 Months				
	CM n=47		UC n=48		*P*	CM n=45		UC n=46		*P*	CM n=45		UC n=45		*P*	CM n=45		UC n=42		*P*
	n	(%)	n	(%)		n	(%)	n	(%)		n	(%)	n	(%)		n	(%)	n	(%)	
Opiate	1	(2)	4	(8)	.36	0		3	(7)	.24	0		2	(4)	.49	0		1	(2)	.48
Hypnotic	1	(2)	2	(4)	1	3	(7)	1	(2)	.36	3	(7)	1	(2)	.61	2	(4)	3	(7)	.67
Antidepressants	8	(17)	5	(10)	.38	9	(20)	8	(17)	.79	12	(27)	6	(13)	.19	8	(18)	8	(19)	1
Pregabaline	2	(4)	2	(4)	1	2	(4)	3	(7)	1	2	(4)	3	(7)	1	1	(2)	2	(5)	.61
Benzodiazepine	3	(6)	2	(4)	.68	3	(7)	4	(9)	1	1	(2)	4	(9)	.36	0		1	(2)	.48

CM: Case Management; UC: Usual Care; IQR: interquartile range
